# Control of Peach Leaf Curl with Foliar Applications of Plant Immunity Inducers and Insights in Elicitation of Defense Responses against *Taphrina deformans*

**DOI:** 10.3390/jof10050325

**Published:** 2024-04-30

**Authors:** Charikleia K. Kavroumatzi, Paschalina Matziarli, Michael Chatzidimopoulos, Anastasia Boutsika, Dimitrios I. Tsitsigiannis, Epaminondas Paplomatas, Antonios Zambounis

**Affiliations:** 1 Hellenic Agricultural Organization-DIMITRA (ELGO-DIMITRA), Institute of Plant Breeding and Genetic Resources, 57001 Thessaloniki, Greece; 2 Laboratory of Plant Pathology, Department of Crop Science, Agricultural University of Athens, 11855 Athens, Greece; dimtsi@aua.gr (D.I.T.);; 3 Department of Agriculture, International Hellenic University, 57400 Thessaloniki, Greece

**Keywords:** crop protection, priming, resistance, leaf curl disease, defense genes, chitosan, yeast cell wall extract

## Abstract

*Taphrina deformans* is the causal agent of leaf curl, a serious peach disease which causes significant losses in peach production worldwide. Nowadays, in order to control plant diseases, it is necessary to adopt novel and low-cost alternatives to conventional chemical fungicides. These promising strategies are targeted at eliciting host defense mechanisms via priming the host through the consecutive application of plant immunity inducers prior to pathogen challenge. In this study, we investigated whether chitosan or yeast cell wall extracts could provide enhanced tolerance against leaf curl in two-season field trials. Furthermore, we addressed the possible molecular mechanisms involved beyond the priming of immune responses by monitoring the induction of key defense-related genes. The efficacy of spraying treatments against peach leaf curl with both inducers was significantly higher compared to the untreated control, showing efficacy in reducing disease severity of up to 62.6% and 73.9% for chitosan and yeast cell wall extracts, respectively. The application of chitosan in combination with copper hydroxide was more efficient in reducing disease incidence and severity, showing efficacy values in the range of 79.5–93.18%. Peach plantlets were also spray-treated with immunity inducers three times prior to leaf inoculation with *T. deformans* blastospores in their yeast phase. The relative expression levels of nine key defense and priming genes, including those encoding members of pathogenesis-related (PR) proteins and hub genes associated with hormone biosynthesis, were monitored by RT-qPCR across three days after inoculation (dai). The results indicate that pre-treatments with these plant immunity inducers activated the induction of genes involved in salicylic acid (SA) and jasmonate (JA) defense signaling pathways that may offer systemic resistance, coupled with the upregulation of genes conferring direct antimicrobial effects. Our experiments suggest that these two plant immunity inducers could constitute useful components towards the effective control of *T. deformans* in peach crops.

## 1. Introduction

Leaf curl, caused by *Taphrina deformans* (Berk.) Tul., is a major disease that has been reported in all peach-growing areas throughout the world [1]. The disease’s severity and the subsequent economic impact on yield may vary depending on the microclimate and the differential resistance responses of the selected cultivars [2,3]. In early spring, when young, developing leaves are affected by the disease, reddish areas appear on them at first; they then progressively become distorted and curl due to cell hyperplasia and hypertrophy [4,5]. Later in spring, when the fungus produces ascospores, severely infected leaves appear reddish-yellow or powdery grey, gradually turning dark brown and abscising prematurely [2]. On unsprayed trees, leaf curl may destroy the first leaves, reducing the crop yield year by year, progressively weakening the trees and reducing their longevity [6]. Infection can also extend to green shoots and fruits; however, differences in disease severity are strongly related to environmental factors [2].

*T. deformans* is a dimorphic ascomycete with both a parasitic and a saprotrophic phase [7]. The pathogen overwinters epiphytically in the buds and wounds or slits of the tree bark in the form of yeast-like blastospores which are dispersed by rain splash all over the tree canopy and cause primary infections in spring [8]. Infection is initiated when blastospores produce hyphae that penetrate the cuticle and invade the plant tissue between the epidermal cells and leaf parenchyma [1]. Foliar anatomy studies revealed that the asci, which are exclusively formed on the upper leaf surface, perforate the cuticle and grow intercellularly, causing leaf curling [4]. Ascospores on the distinctive naked asci of *T. deformans* are formed exclusively during the parasitic phase, whereas the yeast-like phase is saprotrophic and can be grown on artificial media [9]. Ascospores are discharged in spring and initiate the asymptomatic yeast-like saprotrophic phase, during which *T. deformans* is not visually detected as the pathogen constitutes part of the surface mycobiota [10].

Due to the monocyclic nature of the pathogen, one well-timed spray in early spring could efficiently manage the disease [6,8]. However, in practice, even multiple sprays from leaf fall to early blossom may not fully prevent leaf curl in all terminal shoots, which become very susceptible during the early spring period [11]. Cool and wet weather allows the disease to spread into newly emerging leaves, causing severe secondary infections [12]. Particularly in Greece, regular spray programs include two fungicide applications commencing at bud swelling in early spring and two weeks later with various copper formulations (copper hydroxide, copper oxychloride, Bordeaux mixture), ziram, or dodine. However, over the last few years, growers have raised serious concerns regarding the efficacy of the standard spray programs against peach leaf curl, speculating that a decrease in sensitivity to the most commonly applied fungicides may have already emerged.

The introduction of plant immunity inducers and beneficial microorganisms into plant protection systems is a step towards achieving a 50% reduction in the usage of conventional chemical protection products across the EU by 2030 [13,14,15]. Plant immunity inducers are a class of active compounds that can induce systemic acquired resistance (SAR) in plants [13]. Nowadays, chemical and biological inducers can be employed to enhance basal immunity, offering insights into the establishment of novel management solutions for challenging diseases [16,17,18]. The exogenous application of these inducers activates the priming phenomenon, which is an adaptive strategy that prepares plant-specific defense mechanisms for enhanced disease tolerance upon subsequent challenge by pathogens [19,20,21]. During the priming stage, prior to the pathogen attack, a type of immunological memory is provided to the plants, which, coupled with a concurrent induction of gene expression, may be durable [19,22]. Upon the pathogens’ invasion, primed plants effectively mount an accelerated and/or stronger defense response that defines the post-challenge primed state, which results in increased tolerance and in the activation of specific defense-related genes [19,23]. Therefore, priming is an intrinsic part of induced resistance against pathogens, which, in contrast to the direct activation of defense, has lower fitness costs for hosts [21,24]. Chitosan is a known activator of defence responses, acting as an elicitor and a priming agent of systemic resistance in plants [25]. Thus, along with its direct antifungal activity, chitosan’s priming effects result in the suppression of numerous fungal diseases [26,27]. This induced resistance (IR) is often associated with the expression of disease resistance marker genes [28]. Particularly, the priming effects of chitosan are coupled with fine-tune immunity responses, which are highly coordinated, and involve the induction of specific pathogenesis-related (PR) proteins and the synthesis of secondary metabolites with antimicrobial activity [29]. Additionally, the biological control of fungal diseases with antagonistic yeasts as potential biocontrol agents is effective in facilitating the implementation of innovative and environmentally friendly crop protection strategies [30]. Thus, yeasts can trigger systemic defence responses against various pathogens in plants by stimulating the immune and defence priming processes [18]. Particularly, bioproducts based on yeast strains such as yeast cell wall extracts (YCWEs) are used as systemic resistance elicitors inducing a MAMP (microbe-associated molecular pattern)-triggering immunity [28,31,32]. However, the molecular mechanisms behind the activation of systemic resistance upon these treatments remain entirely unknown. Inducers like oligosaccharides, glycoproteins, glycopeptides, and other cellular components are recognized by receptors on the surfaces of plant cells and trigger defence responses, resulting in SAR activation [33]. These complex interactions and their subsequent efficacy on plant pathogens have been determined in various pathosystems [34]. However, little information on the induction of peach resistance and the triggering of defence responses against leaf curl caused by *T. deformans* by plant immunity inducers is available [35,36]. Only recently did pathogen genome sequencing allow for the elucidation of the biochemical and molecular events involved in its interaction with peach [3,37].

Thus, in the present study, we initially evaluated the field efficacy of two plant immunity inducers (elicitors) applied as commercial products of chitosan hydrochloride and cerevisane (cell walls of *Saccharomyces cerevisiae*) against peach leaf curl. Additionally, we addressed the possible molecular mechanisms involved beyond the priming of immune responses by monitoring the induction of key defence-related genes.

## 2. Materials and Methods

### 2.1. Field Trial and Experimental Design

A two-year trial (2022–2023) was conducted in peach (*Prunus persica* L.) orchards (cv. Andross, a susceptible cultivar to leaf curl) located at two different sites in Kavasila (40.589177° N, 22.334929° E) and Arseni (40.702365° N, 22.165320 E°), Central Macedonia, Greece. In 2022, a preliminary trial was conducted at the Kavasila site to verify the possible effects of two plant immunity inducers against peach leaf curl. In 2023, the final evaluation of the two immunity inducers was performed at two different sites; other than Kavasila, an additional trial was established in Arseni because of the increased levels of the disease’s severity noted in that area every year. In all cases, peach trees had been planted in 2015 and pruned to form an open-vase shape at a height of 3.5 m. The soil was silty clay loam in Kavasila and clay in Arseni, containing approximately 2.3% organic matter with a pH of 7.1. A standard insecticide program was followed, and weeds were mechanically controlled using a mower. The trial employed four replications of a randomized complete block design in accordance with the specifications provided by the European and Mediterranean Plant Protection Organization (EPPO) [38]. Each plot consisted of five trees, 4 m apart, arranged in rows that were 4 m apart.

Applications started in mid-February before bud swelling at BBCH-01 [39] and repeated three times every 7 days until flowering in early March (BBCH-60). Sprays were performed using a motorized backpack sprayer (SP 126, Oleo-Mac, Bagnolo in Piano, Italy) with an adjustable 1.4 mm diameter nozzle (S0112000R) at 1000 L ha^−1^. Assessments were conducted on each plot’s three central trees in order to minimize the effects of nearby treatments. The plant immunity inducers evaluated were chitosan hydrochloride (CHI) applied at 3 L/ha (3% *w*/*w* SL, Project One, Phytorgan SA, Athens, Greece), cerevisane (CER) at 0.75 kg/ha (94.1% *w*/*w* WP, Romeo, Hellafarm SA, Athens, Greece), and chitosan hydrochloride in combination with copper hydroxide (CHI-COP) at 3 + 2.1 L/ha (3 SL, Project One, Phytorgan + Heliocuivre 40 SC, Biogard, Athens, Greece). These treatments were compared with two chemical controls (commonly used fungicides against peach leaf curl) which were applied three times according to the label instructions. These were dodine (DOD), applied at 1.65 L/ha (Syllit 544 SC, Arysta LifeScience, Ougrée, Belgium), and copper hydroxide (COP), at 2.1 L/ha (Heliocuivre 40 SC, Biogard). Trees sprayed with tap water only served as untreated controls (CT).

### 2.2. Evaluation of Disease Incidence and Severity in Leaves

Disease incidence and severity were recorded every two weeks on 10 randomly selected marked branches per plot. These were randomly selected from locations all around the trees and at different heights. On each branch, the number of curled and uninfected leaves on 10 young shoots (disease incidence) was counted. The percentage of infected leaf clusters on each shoot (disease severity) in a sample of 100 leaf clusters was also determined. To calculate the area under the disease progress curve (AUDPC), the following formula was used:

$$AUDPC=\sum_{i=1}^{N_{i}-1}\left(\frac{y_{i}+y_{i+1}}{2}\right)\left(t_{i+1}-t_{i}\right)$$

where *t* is the time of each assessment; *y* is the percentage of disease severity at each assessment; and *n* is the number of assessments.

### 2.3. Evaluation of the Tested Products’ Efficacy in Mitigating Leaf Curl Severity

The efficacy of the products was calculated from the severity data using a modification of Abbott’s formula [40]:

$$Efficacy\;(\%)=(1-\frac{S_{T}\;}{S_{C}})\times 100$$

where *S_T_* is the mean severity index for plots in each treatment and *S_C_* is the mean severity index for the untreated controls.

### 2.4. Statistical Analysis

One-way analysis of variance (ANOVA) and Tukey’s honestly significant difference (HSD) post hoc test were used to analyse differences over mean values; *p*-values less than 0.05 were considered statistically significant. Non-normal distributions in percentage and count values were logarithmically transformed to satisfy the assumptions of ANOVA. The statistical package IBM SPSS for Windows, version 25.0 (Armonk, NY, USA: IBM Corp.) was used to analyze the data.

### 2.5. Taphrina Deformans Isolation

In March 2022, peach leaves with visible symptoms of leaf curl harbouring distorted reddish areas were collected from the orchard at the Kavasila site. Isolation of *T. deformans* was conducted using the spore fall method [41] with some modifications. Briefly, ascospores were released from infected circular leaf discs (0.5 cm diameter) into potato dextrose agar (PDA) Petri dishes supplemented with streptomycin. Blastospores were transferred to a yeast malt agar (YMA) medium as previously described [42] and kept in a growth chamber at 22 °C. Upon morphological and microscopical identification, total fungal DNA was extracted from the yeast-phase culture of one representative strain (TdIma1) using the CTAB method. The molecular identification of this strain as *T. deformans* was confirmed through PCR amplification of the rDNA region using ITS1 and ITS4 primers [43]. The sequence of the amplicon was deposited in GenBank (accession no. PP264565).

### 2.6. Pre-Treatment of Plant Material with Immunity Inducers and Pathogen Inoculation

One-year old plantlets of cv. Andross, grown in a greenhouse at 20 to 25 °C with 60% to 70% RH and a 16 h day/8 h night photoperiod, were employed. Young and healthy leaves with non-visible symptoms of natural *T. deformans* infection were sprayed with two plant immunity inducers, chitosan hydrochloride (CHI), and cerevisane (CER). A third treatment consisted of a foliar application of chitosan in combination with a copper hydroxide solution (CHI-COP). Leaves sprayed with sterilized distilled water were used as the control (CT) treatment. All four treatments were applied three times and repeated every 7 days as described previously in field trials. Each treatment was repeated three times using three plantlets per treatment. After each application in all treatments, the leaves were covered with polystyrene bags to prevent any cross contamination.

Immediately after the third application, leaves from all the treatments were detached, transferred to the lab, and inoculated with a *T. deformans* blastospore suspension (9 × 10^6^ blastospores mL^−1^) of strain TdIma1, which was generated by diluting a 4-day-old yeast culture in YMA liquid medium using distilled H_2_O. Each leaf’s abaxial surface was inoculated with 120 μL blastospore suspension under aseptic conditions. For mock-inoculated leaf samples, the YMA medium diluted in distilled H_2_O was spread similarly as the inoculum in previously untreated leaves. The inoculated (TD) and mock-inoculated leaves were sealed inside transparent plastic bags that contained wet filter paper, so that the RH inside the bags was 100%. The bags were kept at 22 °C with 15 h light per day for a 120 h period. Leaf samples were collected at 1, 3, and 5 days after inoculation (dai), frozen in liquid nitrogen, and stored at −80 °C. The experiments were conducted three times, with each replicate containing pooled samples from twelve leaves per each pre-treatment. To confirm the presence of the inoculum in the TD leaf samples, genomic DNA was extracted and a direct molecular detection of *T. deformans* was performed as previously described [9].

### 2.7. RNA Isolation and Gene Expression Analysis

As induced resistance (IR) is coupled with the expression of disease resistance marker genes, in order to determine whether pre-treatments with plant immunity inducers in the greenhouse experiments might have primed their defense-related elicitation in response to subsequent artificial inoculation with *T. deformans*, the relative expression levels of nine marker genes were evaluated using RT-qPCR analysis for all the pre-treatments across the three time points after pathogen challenge. The selected genes had previously been related to induced defense responses against *T. deformans* in peach leaves [3]. These genes encode for members of four different types of pathogenesis-related (PR) proteins, namely PR10 (ribonuclease-like protein), PR12 (defensin 1, DFN1), PR14 (lipid transfer protein 1, LTP1), and PR5 (thaumatin-like protein, TLP1), as well for AOS (allene oxide synthase), PAL (phenylalanine ammonia lyase), ACO (1-aminocyclopropane-1-carboxylate oxidase), ICS (isochorismate synthase), and CAT (catalase) proteins. The list of gene-specific primers is shown in Table S1.

Total RNA was extracted from 100 mg of leaf tissue ground in liquid nitrogen using the Monarch Total RNA Miniprep Kit (NEB, Frankfurt, Germany). RNA quality and quantity were assessed using a NanoDrop spectrophotometer. The LunaScript^®^ RT SuperMix kit (NEB, Europe) was used to synthesize cDNA from the extracted RNA samples using 1 μg total RNA for cDNA library construction. All cDNAs were diluted in RNase-free water (1:8) and stored at −20 °C. The RT-qPCR assays were performed with three technical replicates using the Luna^®^ Universal qPCR Master Mix (NEB, Europe) and the QuantStudio^®^ 5 Real-Time PCR System (Applied Biosystems, Foster City, CA, USA). The total reaction volume was 20 μL and contained 2 μL cDNA, 0.5 μL of each primer (10 μM), and 10 μL Luna Universal qPCR Supermix filled up to the final volume with RNase-free water. The PCR cycling consisted of one cycle at 95 °C for 60 s, followed by 40 cycles at 95 °C for 15 s and 60 °C for 30 s. Melting curve analysis was conducted over a range of 60 °C to 95 °C on all amplified products. The expression profiles of selected genes were normalized through their comparison with a reference gene encoding the elongation factor 1-alpha locus. The relative quantitative expression ratios of TD-inoculated samples compared to mock-inoculated controls were calculated based on the 2^−△△CT^ method employing the RT-qPCR threshold cycle (Ct) values [44]. Gene expression levels are depicted as fold change values for each treatment and are represented as mean values from three independent biological replicates. Statistical analysis of gene expression results was conducted on the logarithmic values of their relative expression in order to ensure that data followed a normal distribution and homogeneity of variances. Statistical differences among treatments are indicated by different letters following a one-way ANOVA analysis and Tukey test for multiple comparisons (*p* < 0.05).

## 3. Results

### 3.1. Evaluation of Field Treatments

In both growing seasons, the first symptoms appeared in untreated plots, approximately two weeks after flowering, in late March. Peach leaf curl infections were recorded in untreated trees (control; CT) in all trials, as indicated by scab severity ratings in the range between 5.23% and 14.23% on leaves, respectively (Table 1 and Table 2). The respective AUDPC values in untreated controls were in the range between 92.69 and 266.69 (Table 1 and Table 2). In late summer, early defoliation symptoms were observed in all trees in the untreated control plots.

In all treated plots, disease incidence and severity were significantly reduced compared with the untreated control on all occasions (*p* < 0.0001). In both seasons, the DOD and COP treatments were of very high efficacy, resulting in minimal levels of leaf curl infection (Table 1 and Table 2). In Kavasila, no significant differences were found between these treatments on both seasons (*p* = 0.29 for 2022 trial; *p* = 0.47 for 2023 trial). The respective efficacy of these fungicides in mitigating disease severity was very high, ranging from 86.1% to 99.8% (Table 1 and Table 2). Likewise, there were no significant differences in disease severity between treatments with CHI, CER, and the CHI-COP combination (*p* = 0.23 for the 2022 trial; *p* = 0.122 for the 2023 trial). However, the efficacy of treatments with the inducers CHI and CER was lower compared that of the CHI-COP combination in both seasons (Table 1 and Table 2). These differences were significant in the 2023 trial (Table 2). Except for one case in the 2022 trial at Kavasila, all defence inducer treatments, including the CHI-COP combination, showed comparable (non-significant) levels of efficacy to those of the fungicides DOD and COP.

In Arseni (2023 season), the disease was more severe, with incidence and severity ratings of 59.5% and 14.23% in the untreated control. As expected, DOD significantly reduced leaf curl infection and showed efficacy at a level of 96.6% (Table 2). The respective AUDPC value for DOD was only 7.27, compared to 266.69 for the untreated control (Table 2). Both treatments with COP (COP and CHI-COP) were less effective compared to DOD, but the differences were not found to be significant (*p* = 0.15). Treatments with the plant immunity inducers CHI and CER were not as effective as the chemical references DOD and COP, showing diseases severity ratings of 5.33% and 4.95% (Table 2). However, their efficacy values reached 62% and 66.9%, respectively. Compared to the untreated control, all treatments, including the inducers, significantly reduced the disease’s incidence and severity in all cases. The AUDPC values of the treated plots were significantly lower in comparison with the untreated control, ranging from 7.27 to 80.07 (Table 2).

### 3.2. Peach Defense-Related Gene Induction by Inducers

To investigate whether inducer treatments using the recommended rates of application tested in field experiments would induce defence genes by priming, one-year-old peach plantlets (cv. Andross) were initially pre-treated three times, a week apart, in the greenhouse. Then, leaves were challenged with the fungus in the yeast phase and tissue samples were collected at specific time points corresponding to certain developmental stages of the disease [3]. Thus, 1 dai represents the stage in which the fungus is still in its penetrating yeast phase, whereas by 3 dai, the transition to its biotrophic phase has been completed, followed by 5 dai, when hyphae growth is well established at the late stage of infection [3].

Particularly, the relative expression profiles of nine key marker genes involved in leaf defence responses were explored after pre-treatment with the two inducers (CER-TD, CHI-TD) or with a combination of chitosan and copper hydroxide (CHI-COP-TD), following artificial challenge with *T. deformans*. Inoculation treatments involving no previous application of inducers were also employed (CT-TD). Relative gene expression levels across all four treatments were assessed in comparison with mock-inoculated leaf samples that had not received any inducer pre-treatment.

**Figure 1:**
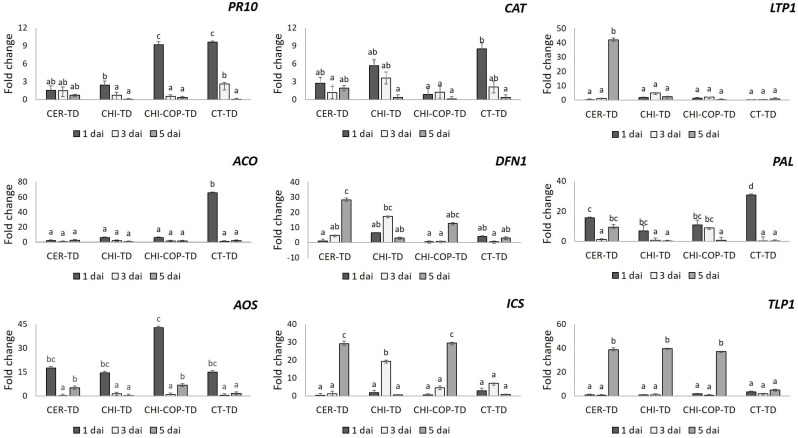
The relative expression patterns of nine key defence-related genes assessed by quantitative real-time PCR in comparison to a peach housekeeping gene (elongation factor 1-alpha). The relative fold change (2^−△△CT^) values of *T. deformans* (TD)-inoculated leaf samples pre-treated with cerevisane (CER-TD), chitosan hydrochloride (CHI-TD), chitosan in combination with copper hydroxide solution (CHI-COP-TD), and untreated (CT-TD) samples were compared to mock-inoculated and untreated (CT) samples. Expression levels are represented as mean values from three independent biological replicates on days 1, 3, and 5 after inoculation (dai). Different letters indicate statistical differences between treatments across all time points after one-way ANOVA analysis and Tukey test for multiple comparisons (*p* < 0.05).

Genes encoding PR proteins showed a differential expression profile which was time-dependent and specific to each treatment (Figure 1). Thus, the relative expression of the *LTP1* gene was significantly and highly increased only on 5 dai in the case of the CER-TD treatment. Similarly, this trend was also retained in the case of the *TLP1* gene at the same time point for all three treatments consisting of inducer applications. In terms of the *PR10* gene, its relative expression was increased early on 1 dai, mainly in the CHI-COP-TD treatment group, and it was also profoundly evident in the CT-TD treatment group. The pattern of accumulation of *AOS* transcripts was consistently induced on 1 dai across all treatments; however, it was more pronounced in the CHI-COP-TD treatment group. While the relative expression levels of the *ICS* and *DFN1* genes were delayed under both the CER-TD and CHI-COP-TD treatments, their expression levels showed an earlier peak on 3 dai in the case of the CHI-TD treatment. Moreover, the *CAT* gene was not significantly induced upon *T. deformans* inoculation in the three inducer treatment groups, except for 1 and 3 dai under the CHI-TD treatment. Notably, the highest relative expression level of this gene occurred early under the CT-TD treatment. Similarly, the *PAL* gene was exclusively induced in the CT-TD treatment group early on 1 dai, and, to a lesser extent, in the three inducer treatment groups at the same time point. It is worth mentioning that the *ACO* gene was highly and significantly expressed only under the CT-TD treatment on 1 dai, whereas under the three inducer treatments, it was almost repressed across the 5-day inoculation period.

## 4. Discussion

The present study provides evidence highlighting the positive impact of two plant immunity inducers (CHI and CER) on the interaction between peach and *T. deformans*. Initially, their effects in terms of reducing disease incidence and severity following foliar sprays were evaluated in efficacy trials under commercial open field conditions on peach trees (cv. Andross) using both untreated and chemical fungicide controls. Furthermore, their efficacy against leaf curl was explored by priming and enabling the induction of leaf defences in response to challenge by the pathogen. Thus, the relative expression of marker genes was monitored at time points corresponding to certain developmental stages of the disease. In field trials, the plant defense inducers were applied three times as alternatives to standard chemical fungicides to simulate standard farming practices. Such applications of CHI and CER, as employed in this study, efficiently protected the peach trees, showing efficacy values from 56.5% to 73.9%. The application of a combination of CHI-COP showed even higher efficacy values, reaching 93.18%. Similar responses to *T. deformans* had previously been reported in peaches after applying several plant growth regulators (alone or in combination) [35]. It is also well postulated that the average field efficacy of plant defense inducers or biocontrol agents against plant pathogens in several host–pathogen systems is lower than the efficacy of the respective chemical compounds [45,46].

The multifunctional role pre- and post-harvest (coating) applications of CHI in horticultural crops has been recently reviewed [47]. The notable efficacy of chitosan against different plant pathogens was previously confirmed in many host–pathogen interactions, including the *Prunus persica*–*Monilia fructicola* pair [48,49]. In grapes, when chitosan was sprayed during the pre-bunch closure and veraison stages, the inhibition of *Botrytis cinerea* growth and the suppression of bunch rot were observed [50]. YCWEs have not undergone practical testing as protective agents in agriculture. Nevertheless, they might function as MAMPs and cause plants to develop resistance against pathogens. Recent studies on foliar CER applications in grapevines against *Plasmopara viticola*, *B. cinerea*, and *Erysiphe necator* revealed the efficacy of this elicitor against downy mildew but not against the other two pathogens [51,52]. Our results showed that CER has a high efficacy against *T. deformans*, which is also an obligate parasite, like *P. viticola*, but differs from it in terms of the infection process. Previously, a reduction in powdery mildew disease symptoms was observed in wheat plants treated with a microbial fermentation mixture [22]. Furthermore, pear fruits treated with cell walls derived from *Rhodosporidium paludigenum* exhibited higher disease tolerance to blue mold rot caused by *Penicillium expansum* [32].

In order to decipher the leaf defense responses and the mechanisms that mediate elevated tolerance to *T. deformans* upon the three pre-treatments with immunity inducers, the expression profiles of nine key defense and priming genes were monitored after leaf inoculation with *T. deformans* blastospores. In terms of the *ACO* gene, which is considered an ethylene biosynthesis marker gene that promotes susceptibility to infection, a common repression pattern was observed across all three time points in the inducer treatment groups, in comparison with its high upregulation (almost 70-fold change) early during the CT-TD treatment. Previously, it had been reported that the *ACO* gene was repressed in resistant peach genotypes upon *T. deformans* inoculation, whereas its transcripts were accumulated in susceptible genotypes [3]. This further highlights the protective priming effect of the three inducer treatments against *T. deformans* challenge. The transcript levels of genes involved in other hormone biosynthetic pathways were also defined in our study. The *AOS* gene, a key regulator in JA biosynthesis and linolenic acid metabolism, was significantly induced, nearly 45-fold, under the CHI-COP-TD treatment at the first time point and to a lesser extent under the other treatments. This gene is induced early in sensitive peach genotypes and is thereafter reduced upon their challenge with *T. deformans*’ yeast phase [3]. It is also known that the *ICS* gene is a hub component for SA biosynthesis, which is thought to be essential for enhancing tolerance to biotrophic pathogens [3]. In our study, the accumulation patterns of *ICS* transcripts revealed a remarkable, almost 30-fold induction under the CER-TD and CHI-COP-TD treatments on 5 dai, as well as an almost 20-fold one under the CHI-TD treatment on 3 dai. These results are consistent with findings concerning *T. deformans* inoculation in a resistant peach genotype, whereas no induction of the *ICS* gene was reported in susceptible genotypes [3]. Our results highlight that SA-dependent defense pathways were also recruited after the three inducer treatments, promoting SAR establishment.

DFN1-encoding genes are involved in host defense responses against pathogens and the relevant proteins inhibit the germination of various fungal pathogens [53]. This gene was postulated to also have functional activity against *T. deformans*, and it was exclusively upregulated in a resistant genotype to *T. deformans* infection, whereas its repression was correlated with susceptibility to the fungus in peach [3]. Notably, and similarly to the relative expression of *ICS* transcripts, the *DFN1* gene was significantly induced in the CER-TD and CHI-COP-TD treatment groupson 5 dai, as well as in the CHI-TD treatment group on 3 dai, when *T. deformans* hyphae started developing in the inoculated leaf tissue. PAL is involved in the induction of phenylpropanoid biosynthesis during fungal infections [54]. This gene was found to be increased in susceptible genotypes earlier and to a lesser extent than in resistant genotypes upon *T. deformans* inoculation [3]. In contrast, our results indicate that the *PAL* gene was universally induced on 1 dai, with significant accumulations of transcripts, particularly in the CT-TD treatment group, followed by the restoration of normal transcript levels at the next time points. This early peak in induction occurs when *T. deformans* is still present in its yeast form in the inoculated leaves [3], while this upregulation is independent of the SA-dependent pathway because *PAL* transcript accumulation occurs before the rise in the *ICS* gene. In contrast, the pattern of accumulation of *TLP1* transcripts was similar in all inducer treatments, tending to increase no earlier than on 5 dai, i.e., during hyphae establishment in the biotrophic phase, consistently with previous reports [3]. This gene, which also exhibits antifungal activity, was induced with a microbial fermentation mixture in wheat seedlings following powdery mildew infection [22]. LTPs, as members of the PR14 protein family, are actively involved in plant–pathogen interactions, acting as positive regulators of plant disease resistance, and may inhibit fungal growth [55,56]. It is worth mentioning that the *LTP1* gene showed a late induction peak only in the CER-TD treatment group. Notably, the relative expression of this gene was dramatically reduced upon *T. deformans* infection in both resistant and sensitive genotypes [3]. The gene encoding ribonuclease-like protein (PR10), which is a member of major peach allergens, was induced across all treatments at the early time point, mainly under the CT-TD and CHI-COP-TD treatments. This gene was previously induced in both resistant and susceptible genotypes upon *T. deformans* challenge [3], which is indicative of its participation in an early defense response. Members of the peach PR10 family have also been linked to decreased susceptibility to *Monilinia* spp. [57]. Finally, the *CAT* gene was mainly induced in the CT-TD treatment group on 1 dai, which might indicate a high ROS accumulation and lipid peroxidation during the hypersensitive response (HR) upon *T. deformans* inoculation.

Even though *T. deformans* is in its biotrophic phase on 5 dai [3], and despite the fact that any defense responses against such pathogens are mediated through a SA-dependent pathway [31], our results indicate that JA-dependent signal transduction pathways were recruited following pre-treatments with both the immunity inducers aiming at eliciting induced systemic resistance (ISR) and during the priming state to mitigate the *T. deformans* challenge. This is remarkably highlighted by the expression profiles of the *DFN1* gene, which is a marker gene for the induction of the JA pathway; this was particularly the case in the CER-TD treatment group on 5 dai. In accordance with this, YCWEs induced the defense response through the activation of *PDF1* in Arabidopsis [31], while previous reports on YCWEs treatments indicated that the genes associated with ISR are induced by such treatments [58]. In addition, other studies indicated that YCWEs, which act as MAMPs and subsequently induce plant defense responses, were effective against both biotrophic and necrotrophic plant pathogens [28]. Particularly, YCWEs were effectively used as elicitors to enhance the tolerance of *Arabidopsis thaliana* and *Brassica rapa* leaves to bacterial and fungal infections by inducing defense genes related to the early triggering of the JA pathway and the late triggering of the SA pathway [28]. Furthermore, YCWEs against *Pseudomonas syringae* pv. *maculicola* and *Colletotrichum higginsianum* appear to act as immunity inducers or activators of plant defense rather than directly acting as bactericides and fungicides [28]. In contrast, a liquid microbial fermentation mixture (a proprietary blend containing bacteria and yeast from fermentation brewing media as well copper sulphate), apart from priming resistance against powdery mildew in wheat, significantly reduced conidia germination at low concentrations [22]. Copper was also reported to trigger defense-related responses in plants [22,59,60]. This suggests that the induction of various defense genes observed in the CHI-COP-TD treatment may also have been influenced by the copper component. A synergistic effect of copper-mediated priming was previously reported in greenhouse trials investigating the synergism of copper fungicides and salicylaldehyde benzoylhydrazone, resulting in a significant reduction in disease severity against the wheat pathogen *Puccinia recondita* [61].

In conclusion, our results indicate that a reduced susceptibility to leaf curl in peach leaves was afforded by both immunity inducers, which were applied separately or in combination, in the case of chitosan with copper hydroxide, as observed in our two-year field trials. This is also supported by our monitoring of key defense genes related to the priming and induction of defense responses against *T. deformans* inoculation. The protective roles of both immunity inducers may be the consequence of a dual action, a direct antifungal response, and the potential of peach leaves to prime their own defense responses to restrict fungal colonization. These results call for further investigation into the specific mechanism of action of these elicitors in order to support their potential application in peach protection against leaf curl disease.

## 5. Conclusions

In this study, we conducted a two-season field trial to investigate whether chitosan and yeast cell wall extracts could improve peach tolerance to leaf curl disease. By observing the induction of key defense-related genes, we also gained insights into the potential molecular mechanisms that might be deployed beyond immune response priming. The efficacy of spraying treatments with both inducers was confirmed. Upon foliar pre-treatment with these plant immunity inducers following *T. deformans* challenge, the relative expression levels of key defense and priming genes were monitored across three time points after inoculation. The results revealed that pre-treatments with these inducers not only triggered the upregulation of genes conferring direct antimicrobial effects, but also activated the induction of genes involved in defense signaling pathways, such as those mediated by SA and JA, which may provide systemic resistance.

## Figures and Tables

**Table 1:** Effects of different spray programs with plant immunity inducers and fungicides against peach leaf curl at the Kavasila site in 2022 ^1^.

Treatments ^5^	Incidence	Severity	AUDPC ^2^	Efficacy on Severity
DOD	1.5 ^3^ ± 0.64 c ^4^	0.11 ± 0.07 c	0.91 ± 0.53 c	98.3 ± 1.2 a
COP	6.5 ± 1.85 bc	0.83 ± 0.24 bc	9.04 ± 2.97 bc	86.1 ± 4.4 ab
CHI	14.75 ± 2.25 b	2.45 ± 0.5 b	39.18 ± 8.93 b	62.6 ± 6.2 c
CHI-COP	11.5 ± 1.47 b	1.38 ± 0.35 bc	18.03 ± 5.16 bc	79.5 ± 4.9 abc
CER	13.25 ± 2.1 b	1.72 ± 0.32 bc	25.59 ± 4.65 bc	73.9 ± 7.7 bc
CT	35.5 ± 4.39 a	6.88 ± 1.1 a	100.43 ± 16.14 a	-

^1^ Data from the last assessment, which was made in late May 2022. ^2^ Area under the disease progress curve was calculated from disease severity scores from four assessments in total. ^3^ Mean values including standard errors of means. ^4^ Means followed by the same letter do not significantly differ (*p* ≤ 0.05, Tukey’s HSD post hoc comparison). ^5^ DOD: dodine; COP: copper hydroxide solution; CHI: chitosan hydrochloride; CHI-COP: chitosan hydrochloride and copper hydroxide solution; CER: cerevisane; CT: control.

**Table 2:** Effects of different spray programs with immunity inducers and fungicides against peach leaf curl at both trial sites in 2023 ^1^.

	Kavasila Trial Site				Arseni Trial Site			
Treatments ^5^	Incidence	Severity	AUDPC ^2^	Efficacy on Severity	Incidence	Severity	AUDPC	Efficacy on Severity
DOD	0.25 ^3^ ± 0.25 c ^4^	0.01 ± 0.01 b	0.08 ± 0.08 b	99.8 ± 0.2 a	2.5 ± 0.29 c	0.44 ± 0.12 c	7.27 ± 2.55 c	96.6 ± 1.3 a
COP	2.75 ± 1.1 bc	0.41 ± 0.16 b	4.08 ± 1.78 b	92.4 ± 3.3 a	16 ± 2.8 bc	2.78 ± 0.51 bc	36.49 ± 6.85 bc	80.6 ± 2.8 abc
CHI	7.75 ± 1.03 b	2.08 ± 0.39 b	32.26 ± 6.57 b	56.5 ± 14.8 b	28.75 ± 3.97 b	5.33 ± 0.99 b	80.07 ± 10.71 b	62.0 ± 6.5 c
CHI-COP	2.25 ± 0.63 bc	0.34 ± 0.14 b	5.01 ± 1.9 b	93.18 ± 2.8 a	16.75 ± 1.25 bc	2.18 ± 0.21 bc	45.02 ± 4.01 bc	84.7 ± 1.6 ab
CER	7 ± 1.35 bc	1.58 ± 0.96 b	16.54 ± 6.37 b	65.9 ± 8.6 ab	25.5 ± 6.96 b	4.95 ± 1.52 b	61.26 ± 16.78 bc	66.9 ± 8.9 bc
CT	21.5 ± 1.56 a	5.23 ± 1.11 a	92.69 ± 7.51 a	-	59.5 ± 2.86 a	14.23 ± 1.36 a	266.69 ± 26.31 a	-

^1^ Data from the last assessment, which was made in late May 2023. ^2^ Area under the disease progress curve was calculated from disease severity scores from four assessments in total. ^3^ Mean values including standard errors of means. ^4^ Means followed by the same letter do not significantly differ (*p* ≤ 0.05, Tukey’s HSD post hoc comparison). ^5^ DOD: dodine; COP: copper hydroxide solution; CHI: chitosan hydrochloride; CHI-COP: chitosan hydrochloride and copper hydroxide solution; CER: cerevisane; CT: control.

## Data Availability

Data are contained within the article and Supplementary Materials.

## Supplementary Materials

The following supporting information can be downloaded at: https://www.mdpi.com/article/10.3390/jof10050325/s1, Table S1: List with the gene-specific primers used in RT-qPCR.
